# Using within-day hive weight changes to measure environmental effects on honey bee colonies

**DOI:** 10.1371/journal.pone.0197589

**Published:** 2018-05-23

**Authors:** William G. Meikle, Niels Holst, Théotime Colin, Milagra Weiss, Mark J. Carroll, Quinn S. McFrederick, Andrew B. Barron

**Affiliations:** 1 Carl Hayden Bee Research Center, USDA-ARS, Tucson, AZ, United States of America; 2 Dept. of Agroecology, Aarhus University, Forsøgsvej 1, Slagelse, Denmark; 3 Department of Biological Sciences, Macquarie University, Sydney, New South Wales, Australia; 4 Department of Entomology, University of California, Riverside, CA, United States of America; RMIT University, AUSTRALIA

## Abstract

Patterns in within-day hive weight data from two independent datasets in Arizona and California were modeled using piecewise regression, and analyzed with respect to honey bee colony behavior and landscape effects. The regression analysis yielded information on the start and finish of a colony’s daily activity cycle, hive weight change at night, hive weight loss due to departing foragers and weight gain due to returning foragers. Assumptions about the meaning of the timing and size of the morning weight changes were tested in a third study by delaying the forager departure times from one to three hours using screen entrance gates. A regression of planned vs. observed departure delays showed that the initial hive weight loss around dawn was largely due to foragers. In a similar experiment in Australia, hive weight loss due to departing foragers in the morning was correlated with net bee traffic (difference between the number of departing bees and the number of arriving bees) and from those data the payload of the arriving bees was estimated to be 0.02 g. The piecewise regression approach was then used to analyze a fifth study involving hives with and without access to natural forage. The analysis showed that, during a commercial pollination event, hives with previous access to forage had a significantly higher rate of weight gain as the foragers returned in the afternoon, and, in the weeks after the pollination event, a significantly higher rate of weight loss in the morning, as foragers departed. This combination of continuous weight data and piecewise regression proved effective in detecting treatment differences in foraging activity that other methods failed to detect.

## Introduction

Hive weight data provide information on the interaction of a colony and its environment with little or no disturbance to the colony. Hive weight changes are a function of several factors, including colony food collection and consumption, bee development and disappearance, moisture gain and loss due to nectar inflow, ambient relative humidity, respiration and drinking [1], as well as robbing, absconding, and swarming. A central goal of researchers employing hive scales has been to characterize these colony events using weight data combined with improvements in sensor technology and analytical approaches [2]. The development of automated sensors for continuous monitoring has allowed researchers to quantify colony metrics beyond daily changes in hive weight due to net food storage and population changes. Linking electronic scales to dataloggers was first reported in 1990, to show hive abandonment to tracheal mite (*Acarapis woodi* (Rennie, 1921)) infestation [3]. Continuous hive weight data have been shown to provide information on weather effects [1,4], swarming [3,4], differences among honey bee races [3], colony growth and consumption [5,6], hive abandonment [7], overwintering [8] and the impact of pesticides on bee colonies [9]. As equipment costs have decreased, recent work has focused on integrating hive scales and other sensors into networks, e.g., [2, 10–12] that facilitate monitoring more hives at the same time and across different locations. As collecting continuous hive data becomes more efficient and more common, exploiting the data thoroughly and efficiently for information about key colony events and performance has become more important.

A key challenge to extracting information about individual colony events from continuous hive weight data is that the raw hive weight represents the sum effect of several contributing factors, including mass loss as the colony respires, consumes food and water, dries nectar, loses bees, and mass gain as the colony forages for nectar, pollen, water, and propolis. While longer-term weight changes mostly involve gains and losses of honey and pollen stores and the bee population, within-day changes largely involve the departure and return of foragers, influx of nectar, pollen and water, and changes in the moisture content of the food and hive parts due to respiration and ambient relative humidity fluctuations. Analysis of within-day weight changes is likely to reveal factors that can change on short notice, such as the size of the foraging population and the availability of forage. Depending on the magnitude of the effect compared to the precision of the scale, within-day weight changes may reveal colony-level effects that are difficult to measure using periodic hive evaluations. To isolate individual factors, within-day weight changes associated with diurnally-reoccurring activities were examined within highly targeted temporal periods. Using this approach, predictable colony events are detected from daily colony mass fluctuations occurring within specific time periods. Successful modeling of factor-associated mass changes depends largely on selection of appropriate monitoring periods and detection of slope changes by regression modeling.

To test our models, we examined continuous hive weight data from colonies subjected to a major colony stressor routinely encountered by honey bees, namely colony malnutrition. Honey bee colonies experience highly variable forage availability, and therefore, colony nutrition, that impacts colony function and survivorship across seasons and landscapes [13,14]. Diagnosing colony malnutrition in honey bee colonies can be difficult since such stress is not due to the presence of a particular compound, but rather the absence or imbalance of one or more nutrients. The effects of nutritional stress have been well described at the level of individual bees and colony outcomes. Individual malnourished workers have smaller HPG, lower protein titers, diminished internal nutrient stores, poorer immune responses, and smaller ovary development than their well-fed counterparts [15–18]. Functionally, nutritionally-stressed workers mature faster into foragers, live shorter lives, and are less efficient at rearing brood, feeding other adults, foraging, and communicating forage locations [13,19–25]. Colonies are known to collectively respond to poor forage and food store availability through modification of colony-level activities (worker and reproductive rearing, foraging, colony defense, temperature management) critical to overall colony performance [26–33]. Despite the centrality of colony nutrition on colony performance, relatively little is known about the colony-level effects leading up to colony malnutrition. Conventional methods of hive assessments may be too infrequent to adequately capture colony responses to nutritional changes. More frequent checks (more than once a week) of colony functions and performance by conventional periodic hive evaluations are impractical both in terms of logistics and colony disturbance. Here, we describe how continuous weight data monitoring can be used to assess changes in colony activities associated with nutritional stress.

## Materials and methods

### 1. CAL 2014 dataset

Eight honey bee hives provided by a commercial beekeeping operation (Hiatt Honey CA LP) were installed in a commercial almond orchard, in four groups of two at regular intervals, on the periphery of a 510 ha irrigated almond orchard near Chowchilla, CA (37° 7'22.25"N, 120°16'21.12"W) in January, 2014, as part of an unrelated study on bee foraging (see [6]). At the start of the experiment, hives consisted of a single deep Langstroth deep box (43.65 l capacity) with 8 frames and a division board feeder. Hive evaluations data (see [6]). In summary, during a hive evaluation each frame was picked up from the box, gently shaken to dislodge adult bees, then weighed and photographed on both sides using a 16.3 megapixel digital camera (Pentax K-01, Ricoh Imaging Co., Ltd.) and replaced in the hive. The area of sealed brood per frame was estimated from the photographs using ImageJ version 1.47 software (W. Rasband, National Institutes of Health, USA). The total weight of the adult bee mass was calculated by subtracting the combined weights of hive components (i.e. lid, inner cover, box, bottom board, frames, entrance reducer, internal feeder) obtained at the start of the experiment from the total hive weight recorded the midnight prior to the inspection. A second box containing 9 frames was added immediately after the hive evaluation on 28 January. Hives were placed on outdoor electronic scales (TEKFA® model B-2418, Galten, Denmark) (max. capacity: 100 kg, precision: ±20g; operating temperature: -30°C to 70°C) and linked to 12-bit dataloggers (Hobo® U-12 External Channel datalogger, Onset Computer Corporation, Bourne, MA, USA) with weight recorded every 15 minutes. Bottom board pollen traps (Sundance bottom mount pollen trap, Brushy Mountain Bee Farm, NC) were placed under each hive at the start of the experiment in January and pollen was collected nearly continuously until 25 March to monitor pollen inflow, after which pollen traps were removed, standard bottom boards installed, and hives evaluated. Hives were then moved 50 km to Madera, CA (36°56'14.09"N, 119°49'52.90"W) as a single group adjacent to fields planted in almond, grape and citrus, and replaced on the hive scales. Hives were evaluated again on 27 May and 20 August. Drought conditions were severe in the area, which impacted available forage.

### 2. SRER 2014 dataset

Hives monitored in the Santa Rita Experimental Range, AZ (31°46'38.08"N, 110°51'47.39"W) [6]. In April 2013, 4 honey bee colonies were established from package bees (approx. 1.5 kg) with Cordovan Italian queens (C.F. Koehnen & Sons, Glenn, CA). The packages were installed in painted, 10-frame, wooden Langstroth deep boxes fitted with migratory wooden lids (Mann Lake Ltd, Hackensack, MN). The apiary was provided with a permanent water source and hives were spaced 1–3 m apart. The hives were placed on scales linked to dataloggers (same models as described above) with weight recorded every 15 minutes. Weight data were collected 11 Feb. to 3 April 2014 and hives were evaluated on 11 March and 3 April 2014.

### 3. CHBRC 2015 dataset

The objectives of this experiment, preventing the departure of foragers for a known period of time at the start of the active period of the day to observe 1) how the rate of weight change during the night was affected by forager departure times; and 2) the timing of any rate change relative to dawn and to control hives. On 29 July 2015 seven honey bee colonies in 10-frame Langstroth deep boxes at the Carl Hayden Bee Research Laboratory, USDA-ARS, Tucson, AZ (32°16'30.17"N, 110°56'28.52"W) were evaluated and placed on hive scales linked to dataloggers as described above. The hives were placed under a shelter with a sun screen that protected the hives from both direct sun (for most of the day) and precipitation. The hives were established from packages in April, 2013, and had been treated with thymol (Apiguard) prior to start of the experiment to control Varroa mite densities. Dataloggers on scales were set to record every minute. Robbing screens (Mann Lake Ltd, cat. no. WW-176) were placed on the entrances of the hives. These screens permit closing the hive entrance without interfering with air exchange. Colonies were divided into two groups, block 1 with 3 colonies and block 2 with four colonies. From 12–28 August, the entrances of all the hives in one block were closed at 5:30AM, i.e. before daybreak and the start of flight activity. Closed entrances were opened after a pre-determined period of time: 1 h, 1.5 h, 2 h, 2.5 h or 3 h (S1 Table). Entrances for a given block were closed only on alternate days, and closures were conducted for 4–5 days per week. Resulting within-day weight data were analyzed using piecewise regression, using 100 iterations of regressions with 4 break points, to compare planned and observed delays with respect to data parameters.

### 4. MU 2017 dataset

The objective of this experiment was to determine the extent to which the slope of the piecewise regression segment associated with morning forager departure is affected by bee movement (arrivals-departures). In November 2016 16 honey bee colonies established in 10-frame Langstroth deep boxes at Macquarie University, Sydney, Australia (33°46'27.91"S, 151° 6'46.23"E) were placed on hive scales linked to dataloggers as described in the sections above. The dataloggers were set to record every minute. On 29 March and 20, 21 and 24 April hive entrances were recorded on video for one minute per hive on each of four hives. A different set of four hives was chosen each day, so each hive was monitored once. The videos were then analyzed frame by frame with the software Cowlog 3.0.2 to record all bee departures and arrivals during that minute, which occurred between 8:49 to 8:59 AM depending on the day. Piecewise regression models with 4 and 5 break points were fit to the detrended within-day weight data, and the net change in bee number was regressed on slopes of the segments, from 100 iterations of regressions with 5 break points, for that time period during which the observations were made.

### 5. CAL 2015–16 dataset

In November 2015 32 honey bee colonies that occupied one to two 10-frame Langstroth deep boxes and had marked queens were identified in apiaries in southern Arizona (see [34]. The hives were divided into four groups of 8. Six hives in each group, for a total of 24 hives, were placed on electronic scales (same scales and dataloggers as described in the previous section). All hives were given 250 g protein patty supplement at the end of November, evaluated to determine adult bee mass in the first week of December, and fed both protein patty supplement and 3 L of 1:1 sugar syrup in mid December. Hive evaluations were conducted on all hives as described in the previous section. On 30 December the four groups of eight hives were moved to one of four sites: two sites near Red Rock, AZ (32°33'13.75"N, 111°19'13.76"W), hereafter “RR” and two sites at the University of Arizona’s Maricopa Agricultural Center in Maricopa, AZ (33° 5'16.02"N, 111°58'45.19"W), hereafter “MAC”. The two hives in each group not on hives scales were fitted with front loader pollen traps (Brushy Mountain, Moravian Falls, NC, USA) to monitor foraging success. Colonies at the RR sites had access to ample forage provided in a plot of *Brassica rapa* and also foraged on *Encelia farithreenos*a, *Larrea tridentata*, *Searsia lancea*, and *Erodium* spp., while colonies at the MAC sites had access to about a third as much forage. Hives were again evaluated on 26–29 January and on 2–4 February all colonies were moved to Blackwell’s Corner, CA (35°38'37.28"N, 119°54'56.30"W) where they were placed in an almond grove prior to bloom and fed sugar syrup. On 5 March all colonies were evaluated and then moved to a holding yard at Keck’s Corner, CA (35°40'10.69"N, 120° 6'17.15"W), where they were fed sugar syrup. All surviving hives were evaluated for a final time on 4–8 April 2016 and then moved back to Arizona. As the study proceeded, hives that died were replaced by other hives from the same treatment groups; statistical analysis reflected that.

### Data analysis

Hive weight data were detrended for each day by subtracting the weight estimate of the hive between midnight (or closest time thereafter) from each subsequent weight value during the day until the last value just before midnight. This daily dataset had three main parts: the period from midnight until about dawn, when colony’s interactions with the environment in terms of weight were limited to temperature effects and gas exchange (e.g., water vapor), the active period during the day from the initial departure of bees about dawn until their return about dusk, and the period from dusk to midnight when the colony was again quiescent. In the CAL 2015–16 field experiment, hive weight data were also analyzed using the method previously described (see [6]): data were detrended by subtracting raw data from the hourly 25 hour running average, sine curves were fit to the resulting data, and the amplitudes of those curves were used as a response variable (reflecting bee flight activity).

Within-day weight changes were modeled using the “segmented” function in R [35] which fits a segmented line derived from linear or generalized linear model to a dependent variable. The number of breakpoints can be estimated automatically or through the use of initial values provided by the user with final determination by the function. Using the segmented function to automatically determine the number of break points tends to overestimate the number of break points so estimation via methods such as visual inspection is preferred for the final model [35]. Function outputs include the final break point values, the slopes and intercepts of the segments, and the overall model fit. An effort was made to use a minimum number of break points to ensure the model did not have an excessive number of arbitrary parameters but did reflect changes in weight with sufficient precision. Initially models with three break points were fit: break point 1—estimated sunrise, which was calculated with respect to day of year, latitude and longitude; break point 2—minimum weight value of dataset; and break point 3—estimated sunset, using the same function as for sunrise. Models with one and two additional break points were also considered, with those initial break point values estimated at regular intervals during the active part of the day. Model output was visually compared to raw data. Our intention was to determine a suitable general model, rather than the optimal model for each day. Parameters for the model with the highest adjusted r^2^ value out of 100 iterations were retained.

## Results

### Detrending hive weight data

**Fig 1:**
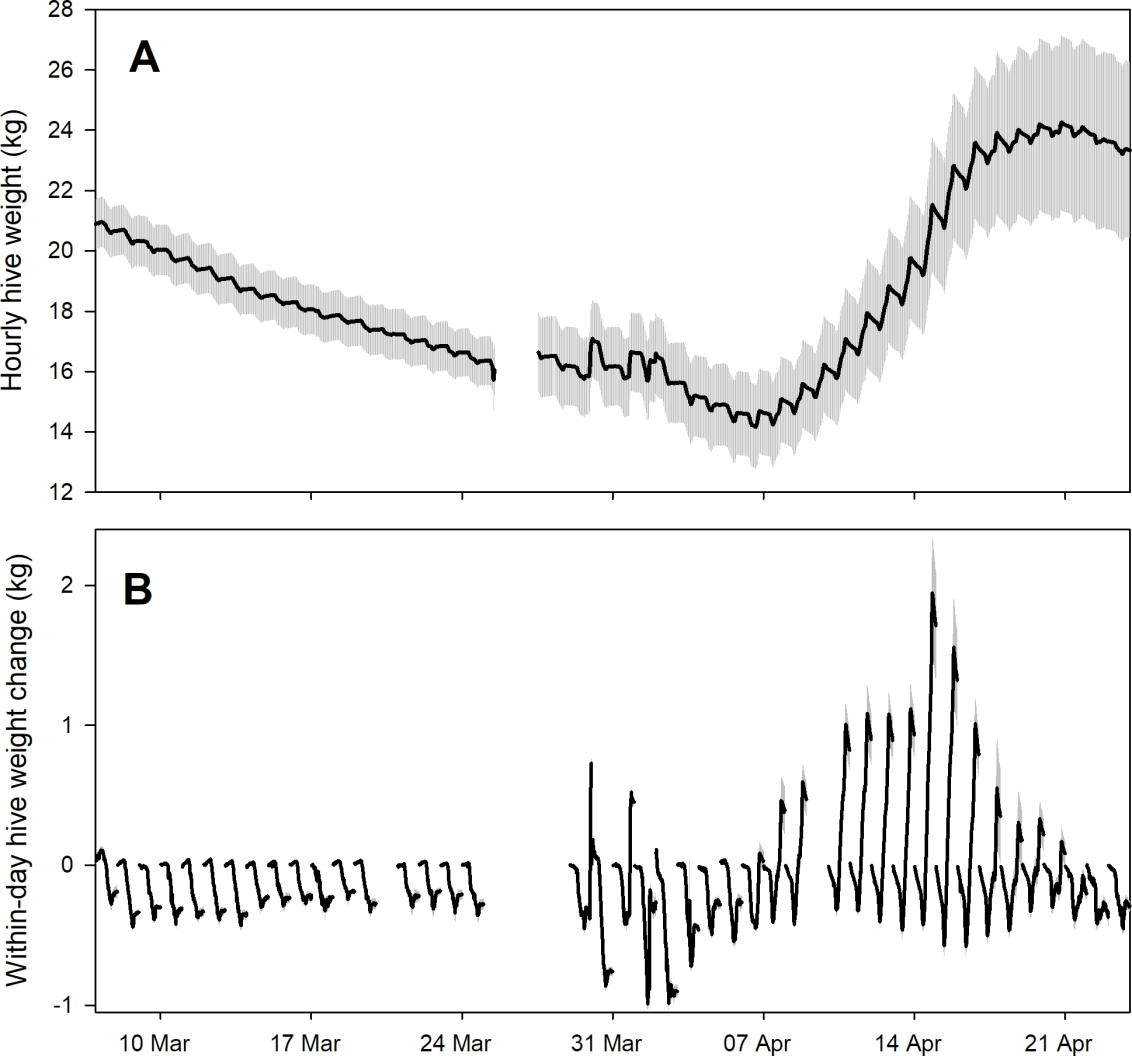
Average (± s.e.) hive weight and within-day weight changes for 8 honey bee hives kept near Madera, CA (the CAL 2014 dataset). Data are shown from 7 March to 23 April 2014. A) raw data; B) within-day weight changes.

**Fig 2:**
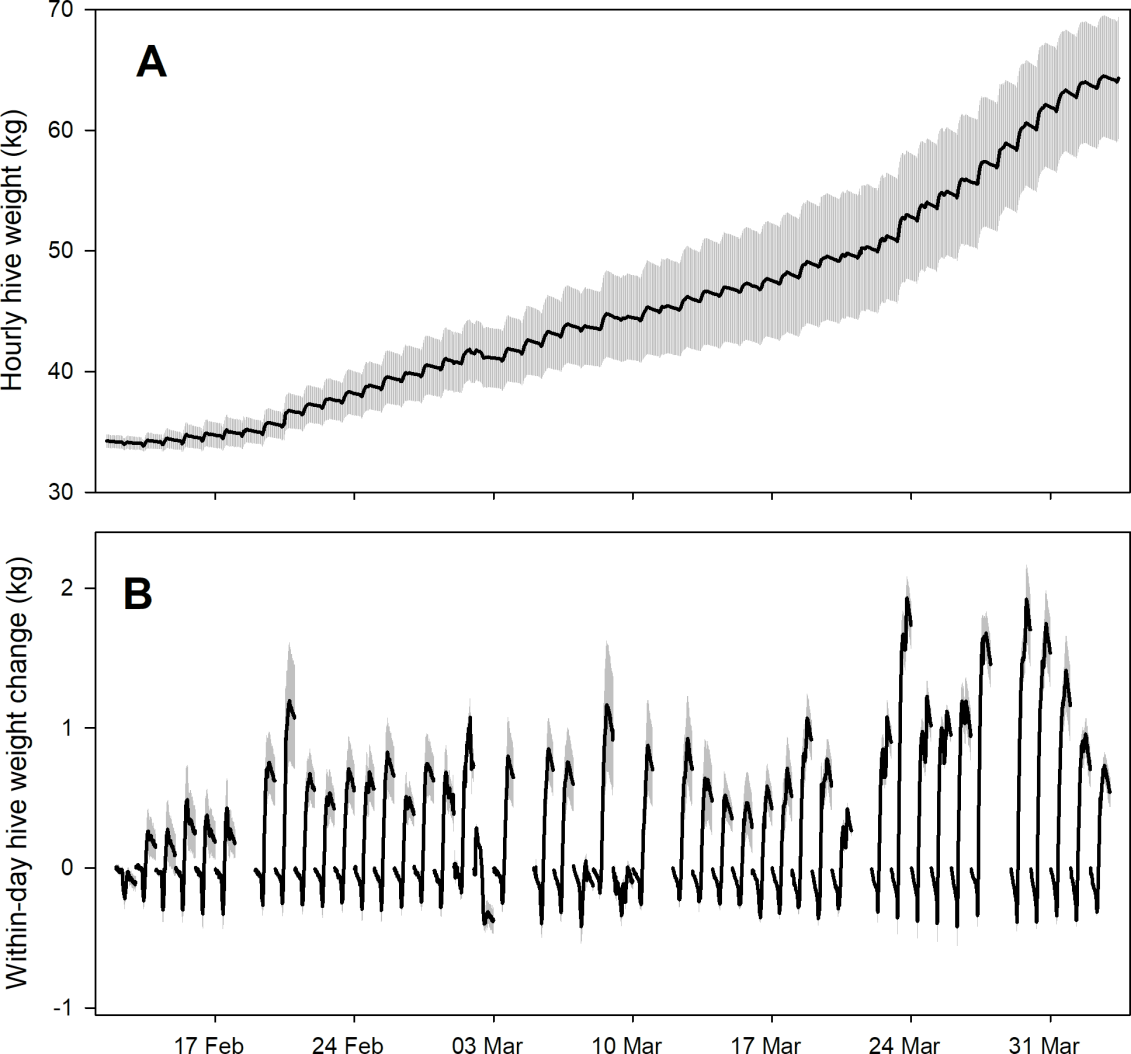
Average (± s.e.) total hive weight and within-day weight changes for 3 honey bee hives kept near Green Valley, AZ (the SRER 2014 dataset). Data are shown from 11 February to 2 April 2014. A) raw data; B) within-day weight changes.

Detrending data by subtracting hive weight at midnight yielded consistent patterns among hives over time (Figs 1 and 2). CAL 2014 data showed colony weight loss in mid-winter followed by a nectar flow in March with an ensuing dearth. SRER 2014 data showed colony weight gain during a late winter to early spring nectar flow. While the bee colonies themselves differed in size (Table 1), after detrending the data by subtracting the hive weight at midnight, the within-day patterns of weight changes were largely consistent over periods of time that corresponded to nectar flows and other events.

**Table 1:** Adult bee masses and capped brood areas (averages ± s.e.) for all sampling occasions of all datasets used in this study. N refers to the number of hives in that treatment group remaining from the original hives on scales at the start of the experiment.

Data set	Group	N	Date	Adult bee mass (g)		Capped brood area (cm^2^)	
				average	s.e.	average	s.e.
CAL 2014		8	1/29/2014	2034	±73	-	-
		8	3/25/2014	2010	±154	1773	±62
		8	5/27/2014	3488	±210	2227	±140
		3	8/20/2014	2008	±1000	979	±880
SRER 2014		3	3/11/2014	2606	±284	2946	±244
		3	4/3/2014	4231	±911	4667	±249
CHBRC 2015	Block 1	3	7/29/2015	1494	±492	1343	±352
		3	8/28/2015	1291	±453	917	±275
	Block 2	4	7/29/2015	1443	±153	1438	±148
		4	8/28/2015	1417	±58	1014	±178
CAL 2015–16	MacN	6	12/7/2015	1246	±205	417	±141
		6	1/28/2016	1218	±70	101	±25
		3	3/3/2016	680	±74	479	±11
		3	4/5/2016	854	±418	1115	±711
	MacS	6	12/7/2015	1378	±218	561	±119
		6	1/28/2016	1557	±171	191	±76
		3	3/3/2016	1001	±94	405	±274
		3	4/5/2016	582	±190	928	±838
	RREast	6	12/7/2015	1185	±168	622	±87
		6	1/28/2016	1729	±255	186	±93
		5	3/3/2016	888	±74	501	±150
		5	4/5/2016	763	±234	782	±348
	RRWest	5	12/7/2015	1608	±341	404	±129
		5	1/28/2016	1611	±142	133	±30
		5	3/3/2016	935	±156	854	±224
		5	4/5/2016	996	±195	1409	±243

### Identifying break points

**Fig 3:**
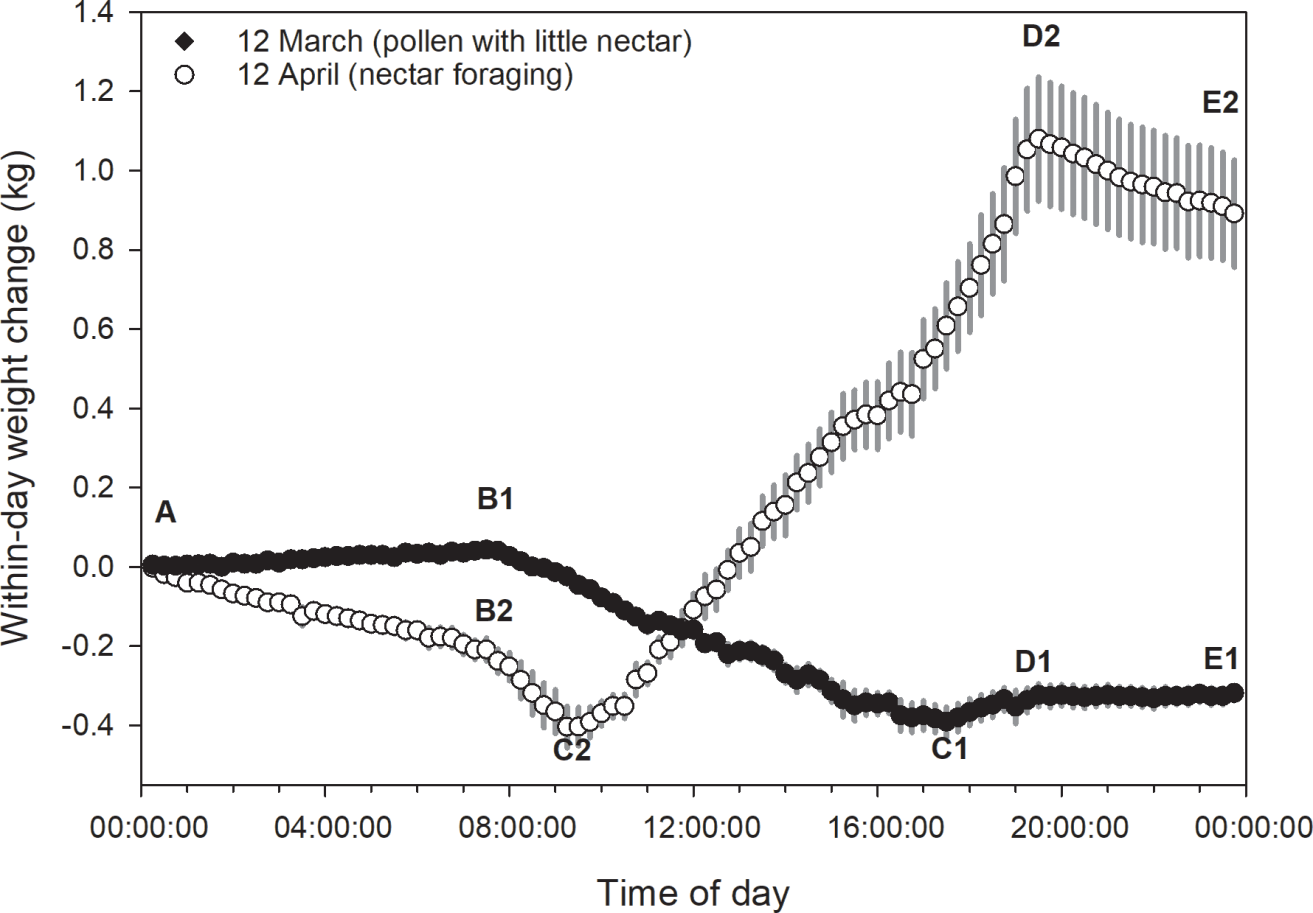
Examples of two within-day weight change patterns obtained from average (± s.e.) 15-minute weight data from 8 hives kept near Madera, CA (see Fig 1) (the CAL 2014 dataset). See text for details.

Most daily activity patterns can be considered as having 4 to 6 segments (Fig 3). Two examples of daily patterns with 3 break points were shown with the following interpretations:

Point A: First weight measure at midnight or shortly thereafter;
Segment AB: Inactive period in the early morning; hive weight change is likely due to bee respiration and changes in the moisture content of nectar, pollen and wooden hive parts;
Point B: Departure of foragers and other bees at beginning of active period (usually close to dawn if temperatures permit);
Segment BC: Active period usually showing hive weight loss due to greater numbers of departing bees compared to returning bees;
Point C: Point at which mass of returning foragers plus nectar and pollen exceeds the mass of departing bees plus weight loss due to drying and respiration in the colony;
Segment CD: Active period usually showing hive weight gain due to returning foragers;
Point D: Return of bees to the hive around dusk is completed;
Segment DE: Inactive period with hive weight change driven mainly by respiration and changes in ambient humidity–usually close to parallel with segment AB;
Point E: Last weight measure just before midnight.

### Optimizing the number of model break points

In most locations a hive is active, when ambient temperatures permit, from about dawn to about dusk. Few if any foragers depart a hive before dawn or return to the hive after dusk. An initial break point before dawn would therefore probably correspond to a weight change caused by something other than initial hive activity, such as rainfall. Similarly, a break point after dusk likely corresponded to an event other than forager return. Considering break points falling between 8PM and 4AM as errors a model with 3 break points had the fewest, followed models with 4 and 5 break points.

CAL 2014 data were averaged over 8 hives and the SRER 2014 data were averaged over 3 hives. Those average values were then modeled using regression lines with 3, 4 and 5 break points. The Hiatt 2014 dataset was divided into a period of hive weight loss with comparatively little nectar (29 January to 24 March, which included almond pollination) and a period of citrus nectar flow (29 March to 26 May). Five parameters were compared among the different models: the average adjusted r^2^ for each fit, the number of days in which any fits were successful, the number of successful fits out of 100 iterations for those days where fits were successful, and number of “errors” for the 1^st^ and last break points, with a 1^st^ break error defined as a break before 4AM (i.e. before bees start foraging) and a last break error defined as a break after 8PM (i.e. after bees would have returned to the hive) (Table 2).

**Table 2:** Comparison of piecewise regression model fits using different numbers of break points across different datasets. Shown are average values ± s.e. 1^st^ break point errors were considered initial break points that occurred before 4AM and last break point errors were considered final break points that occurred after 8PM.

Data set	No. break points	Adjusted r^2^ (average ± s.e.)		Percentage fits per day (average ± s.e.)		Percentage of days fit	1^st^ break point errors	Last break point errors
CAL 2014	3	0.978	±0.400 a	94.7	±1.6 a	87.3	5	1
29 Jan.-24 Mar.	4	0.984	±0.427 ab	79.4	±3.4 b	89.1	5	2
	5	0.993	±0.103 b	61.9	±3.8 c	74.5	5	2
CAL 2014	3	0.960	±0.500 a	98.2	±0.7 a	93.2	2	11
29 Mar.-26 May	4	0.974	±0.346 b	85.0	±3.3 b	93.2	3	12
	5	0.985	±0.230 b	77.0	±3.1 b	88.1	4	13
SRER 2014	3	0.986	±0.005 a	94.1	±2.2 a	90.0	1	0
12 Feb.-3 Apr.	4	0.993	±0.003 ab	91.0	±2.7 a	90.0	1	1
	5	0.995	±0.002 b	73.7	±2.9 b	90.0	2	0

Regressions with 3 break points had significantly lower adjusted r^2^ values on average but successfully fit more iterations, for those days where any 3 break point regressions could be fit, than regressions with 5 break points, while regressions with 4 break points had r^2^ values equal to or greater than models with 3 break points and to fit as many or more datasets than models with 5 break points. That hives tended to change weight at rates that were largely constant for sufficient periods of time, often up to several hours, and that the average r^2^ values of successful fits equaled or exceeded 0.96, suggested that the piecewise regression approach is appropriate.

**Fig 4:**
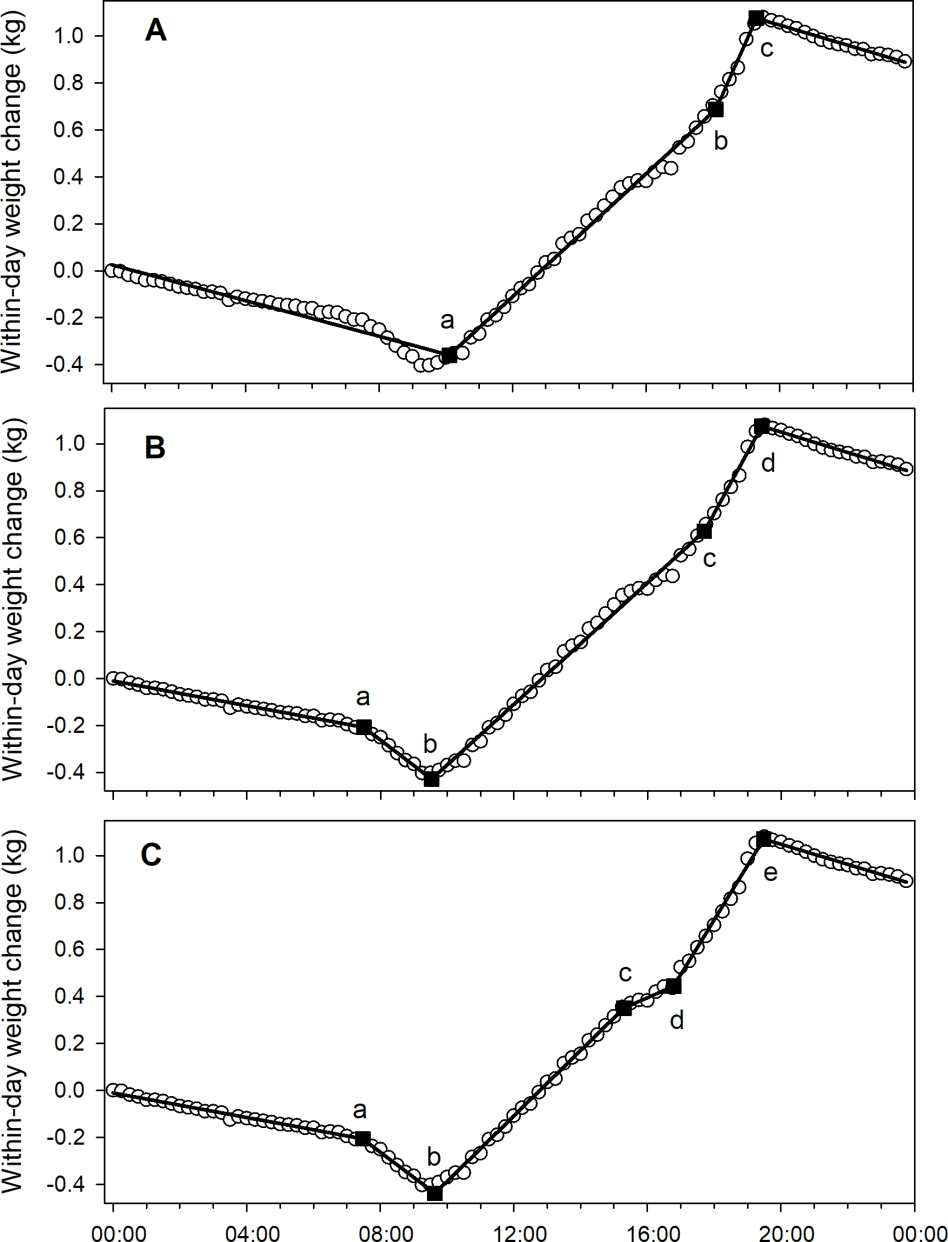
A sample day data subset (April 12, 2014, the same subset as Fig 3; from the CAL 2014 dataset) fit with piecewise regression models with different numbers of break points. A) 3 break points (r2 = 0.9968); B) 4 break points (r2 = 0.9987); and C) 5 break points (r2 = 0.9993). Solid line show regression line, and labelled points a, b, c, d and e (solid squares) show break points 1–5 with a = 1st break point, b = 2nd break point, etc. Empty circles show within-day weight change data.

Data for April 12, 2014, in the CAL 2014 dataset, as shown in Fig 3, were fit with 3, 4 and 5 break point regression models (Fig 4).

**Fig 5:**
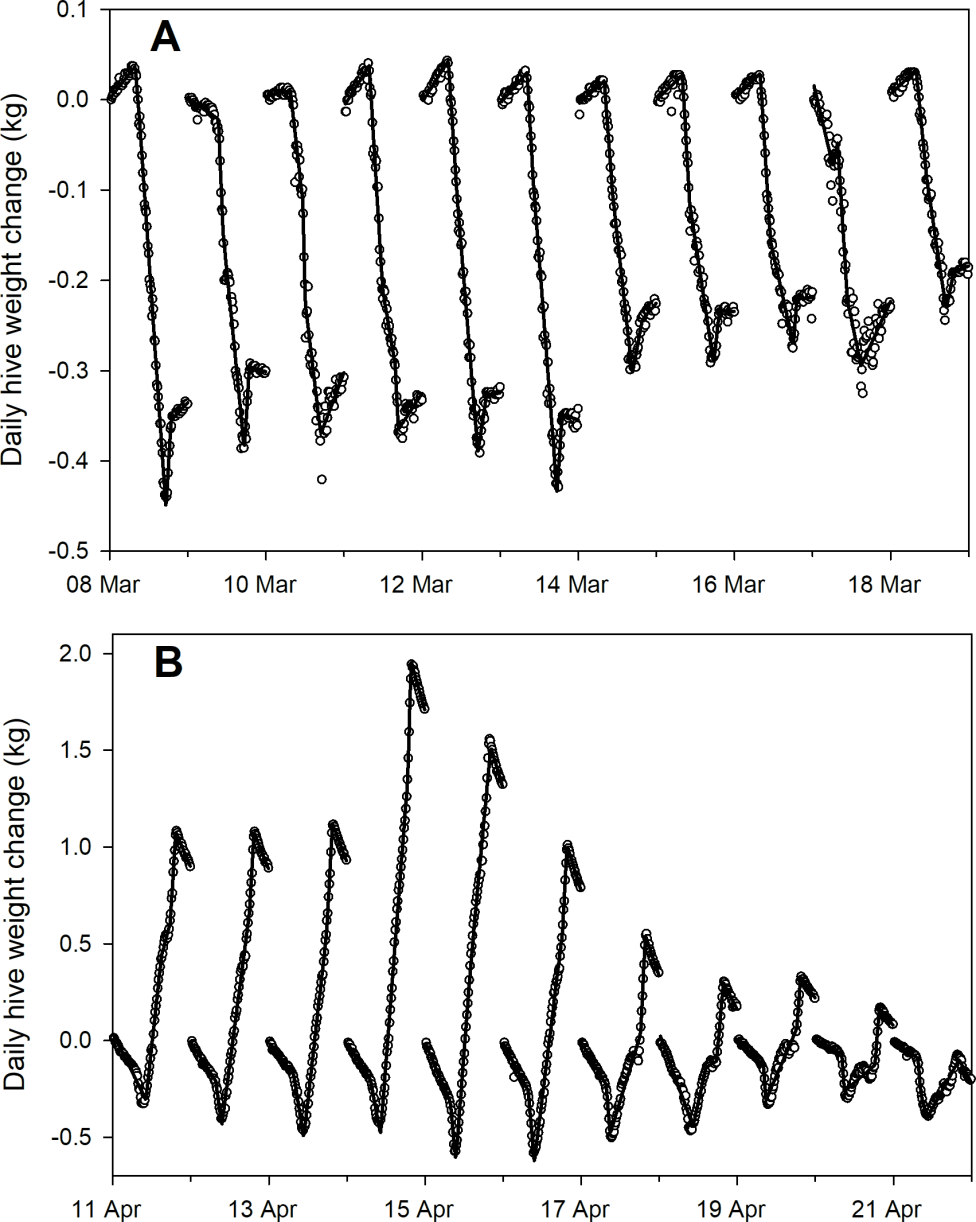
Piecewise regression curves, with 4 break points fit to average within-day hive weight changes from 8 hives kept near Madera, CA (the CAL 2014 dataset). A) Data from 8–18 March 2014 (little or no nectar flow); B) 11–21 April (citrus nectar flow within foraging distance).

That particular dataset illustrated another kind of error that would be difficult to correct: the function fails to detect a break point associated with early departure that can be detected through visual inspection. In this case models with 4 and 5 break points detected the departure while the model with 3 break points did not. Models with 4 break points fit to data samples showed an adequate fit (Fig 5).

### Interpreting model parameters

**Fig 6:**
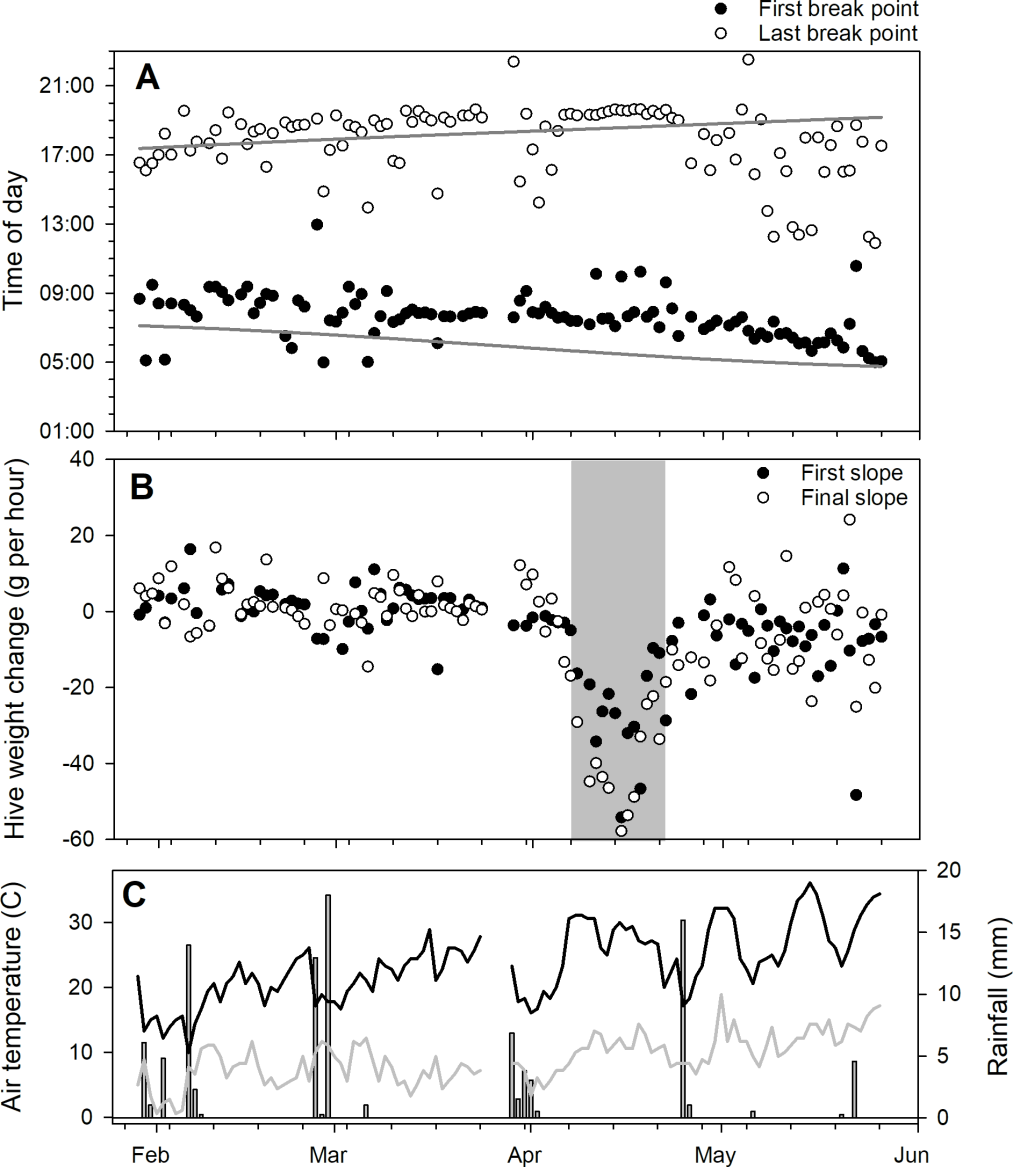
Examination of parameters from 4-break point piecewise regression models fit to average within-day hive weight changes for 8 honey bee hives kept near Madera, CA from 29 Jan. to 26 May 2014 (the CAL 2014 dataset). A) 1^st^ and 4^th^ breakpoints for piecewise regression curves. In cases where the 1^st^ breakpoint occurred before 4AM (i.e. before dawn) the 2^nd^ breakpoint was used. Likewise, if the 4^th^ breakpoint occurred after 8:00PM the 3^rd^ breakpoint was used. Gray lines show local sunrise and sunset. B) 1^st^ and 5^th^ segment slopes for piecewise regression models fitted to average within-day hive weight changes for the same hives. Gray zone indicates a period of nectar flow. C) Rainfall and minimum and maximum temperature data.

**Fig 7:**
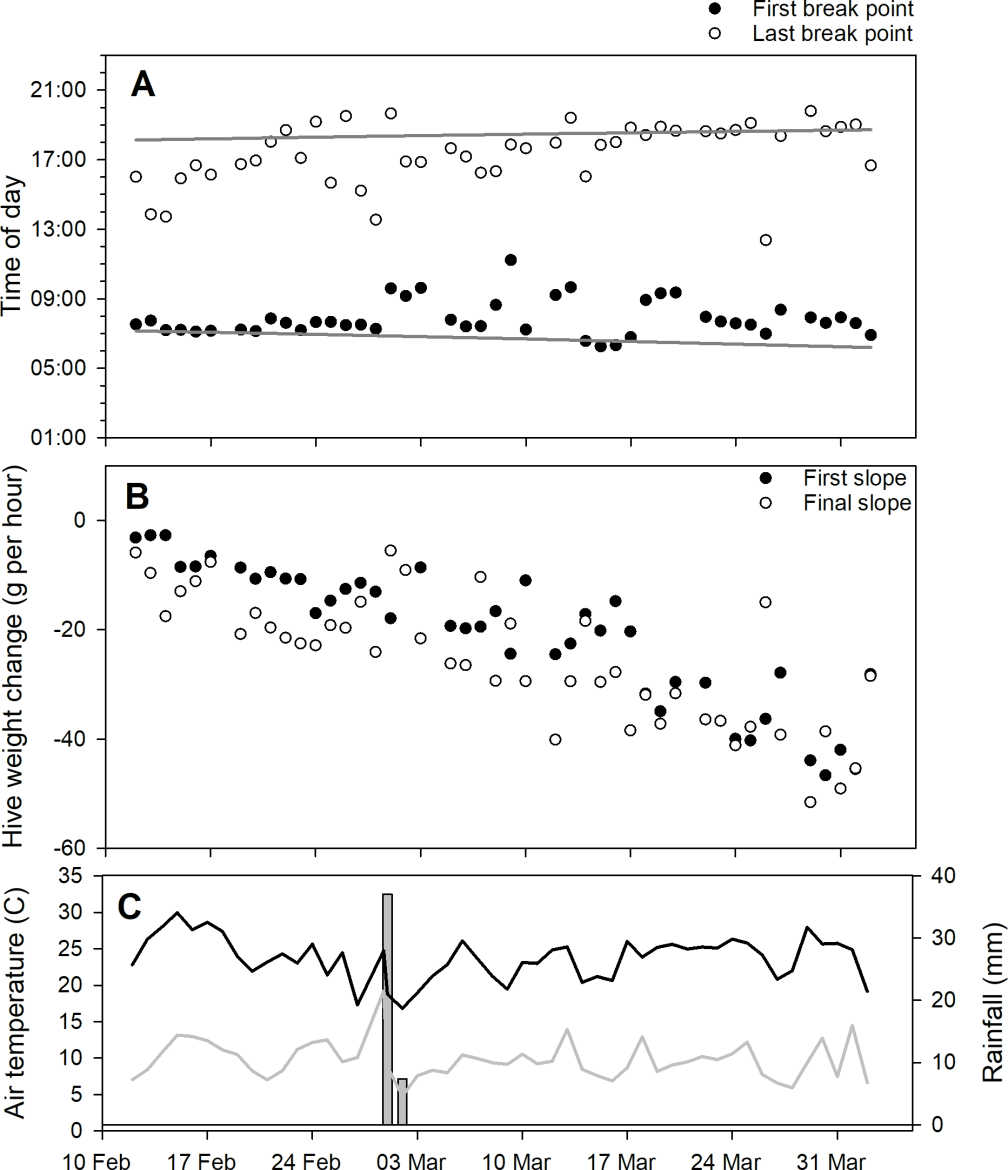
Examination of parameters from 4-break point piecewise regression models fit to average within-day hive weight changes for 3 honey bee hives kept near Green Valley, AZ, from 11 Feb. to 2 April 2014 (the SRER 2014 dataset). A) 1st and 4th breakpoints for piecewise regression curves. In cases where the 1st breakpoint occurred before 4AM (i.e. before dawn) the 2nd breakpoint was used. Likewise, if the 4th breakpoint occurred after 8:00PM the 3rd breakpoint was used. B) 1st and 5th slopes for piecewise regression curves fitted to average within-day hive weight changes for those same hives. C) Rainfall and minimum and maximum temperature data.

Models with 4 break points were determined to have the best fit and used in subsequent analyses. Models fit to data from the CAL 2014 and SRER 2014 datasets, and the break point and slope values changed from day to day (Figs 6 and 7).

Forager departure and return estimates tended to diverge through the winter and spring, as would be expected as daylight hours and ambient temperatures increased. Hive weight changes during the inactive period at night were influenced by the presence of fresh nectar. A nectar flow was present throughout the SRER 2014 data set but a discrete, short-term citrus nectar flow was observed in the CAL 2014 dataset. Hives tended to lose weight at night during a nectar flow, probably due to moisture loss from drying nectar, but outside of a nectar flow hives usually maintained or even gained weight, probably because of increasing moisture content of hive wooden parts as the ambient relative humidity rose at night. Meikle et al. (2006) observed that hourly weights of empty hives varied by 50 g or more over 24 hours.

### Manipulating initial bee departure

Visual examination of the data showed that while the early morning weight curves were generally maintained when the bees were prevented from leaving, some small, additional weight changes were observed, often around the time of the 1^st^ break point for the control colonies (S1 Fig). The exact cause of those changes was unclear. A comparison of planned vs. observed changes in the first break point showed a significant linear relationship (F_1,13_ = 73.06, p<0.001, adj. r^2^ = 0.85; Shapiro-Wilk normality test and constant variance test both passed) (S2 Fig). The slope of that line was 0.649; that the slope is not equal to one indicates that there were factors other than bee departure, such as moisture gain or loss due to changing ambient conditions, influencing the break points.

### Correlating bee traffic with regression slopes

Piecewise regression lines with 5 break points fit data had a higher r^2^ (0.987±0.002) and fit the data more often (89.6±4%) (S1 File) than those with 4 break points (0.976±0.005 and 82.5±5%, respectively). The slope of the regression segment fit to weight data collected during the time of the observation was regressed on the net movement of bees (arrivals-departures) at that time (S3 Fig). Net movement was significantly correlated with hive weight change, as measured by the segment slope (F_1,13_ = 26.66, p = 0.0002, adj. r^2^ = 0.65; Shapiro-Wilk normality test and constant variance test both passed), indicating that bee traffic was a significant component of hive weight change at that time. The absolute value of the regression slope, 0.032 g loss per departing bee, reflects the greater per capita mass of returning foragers, laden with nectar and pollen, compared to departing bees. Assuming all the hive weight change at that point in time was due to bee traffic, that change can be expressed as:

$$\Delta W=\frac{N_{D}M_{D}-N_{A}M_{A}}{N_{D}-N_{A}}$$ [1]

where Δ*W* is the hive weight change (slope of the regression segment, here 0.032g/min), N_D_ and N_A_ are the numbers of departing and arriving bees, respectively, and M_D_ and M_A_ are the masses of departing (unladen) and arriving (laden) bees, respectively. Assuming M_D_ = 0.13 g [5], using data on N_D_ and N_A_, and rearranging the terms, the average mass of an arriving bee, M_A_ would have been about 0.15 g.

### Applying within-day analysis to migratory colony nutrition study

The two treatment groups were not significantly different in terms of adult bee mass or brood surface area (see Table 1) during any hive evaluations. Continuous hive weight data were divided into three parts: before, during and after almond pollination. Of the 17 colonies in the low-forage treatment just prior to shipping to almond pollination on 28 January, only 8 (47%) survived to the last evaluation on 5 April, but of the 19 colonies in the high-forage treatment, 14 (74%) were still alive for the final evaluation.

Each hive weight dataset was subjected to a sine curve analysis of 3-day subsets of detrended data [6] and the resulting curve amplitudes were compared between treatments using repeated measures MANOVA. Amplitude data from every 3^rd^ day were used, to avoid overlap between data subsets. No treatment differences were detected with respect to sine amplitudes for any of three datasets (pre-almond pollination: P = 0.946; during almond pollination: P = 0.274; post-almond pollination: P = 0.998).

Piecewise regression analysis yielded estimates for 10 parameters: 4 break point values, 5 slope values and the adjusted r^2^. Because the data were detrended by subtracting the raw data value at midnight, daily datasets were mathematically independent. A repeated measures MANOVA was conducted on these daily parameter values of interest:

1. time of initial forager departure (break point nearest dawn, usually the 1^st^);
2. time of final forager return (break point nearest dusk, usually the 4^th^);
3. slopes of the 1^st^ segment and 5^th^ segment (associated with weight changes at night when colonies are not foraging);
4. slope of the first segment after initial forager departure, usually the 2^nd^ segment (rate of weight loss largely due to forager departure); and
5. slope of the last segment before dusk, usually the 4^th^ segment (rate of weight gain due forager return).

For statistical analysis, if the 1^st^ break point occurred before 4AM, the 2^nd^ break point was used as the time of initial forager departure (with no restrictions placed on that second estimate) and the slope of the 3^rd^, rather than 2^nd^, segment was used as the rate of weight loss due to forager departure. Likewise, if the 4^th^ break point occurred after 8PM then the 3^rd^ break point was taken as the time of final forager return (with no restrictions placed on that second estimate).

**Fig 8:**
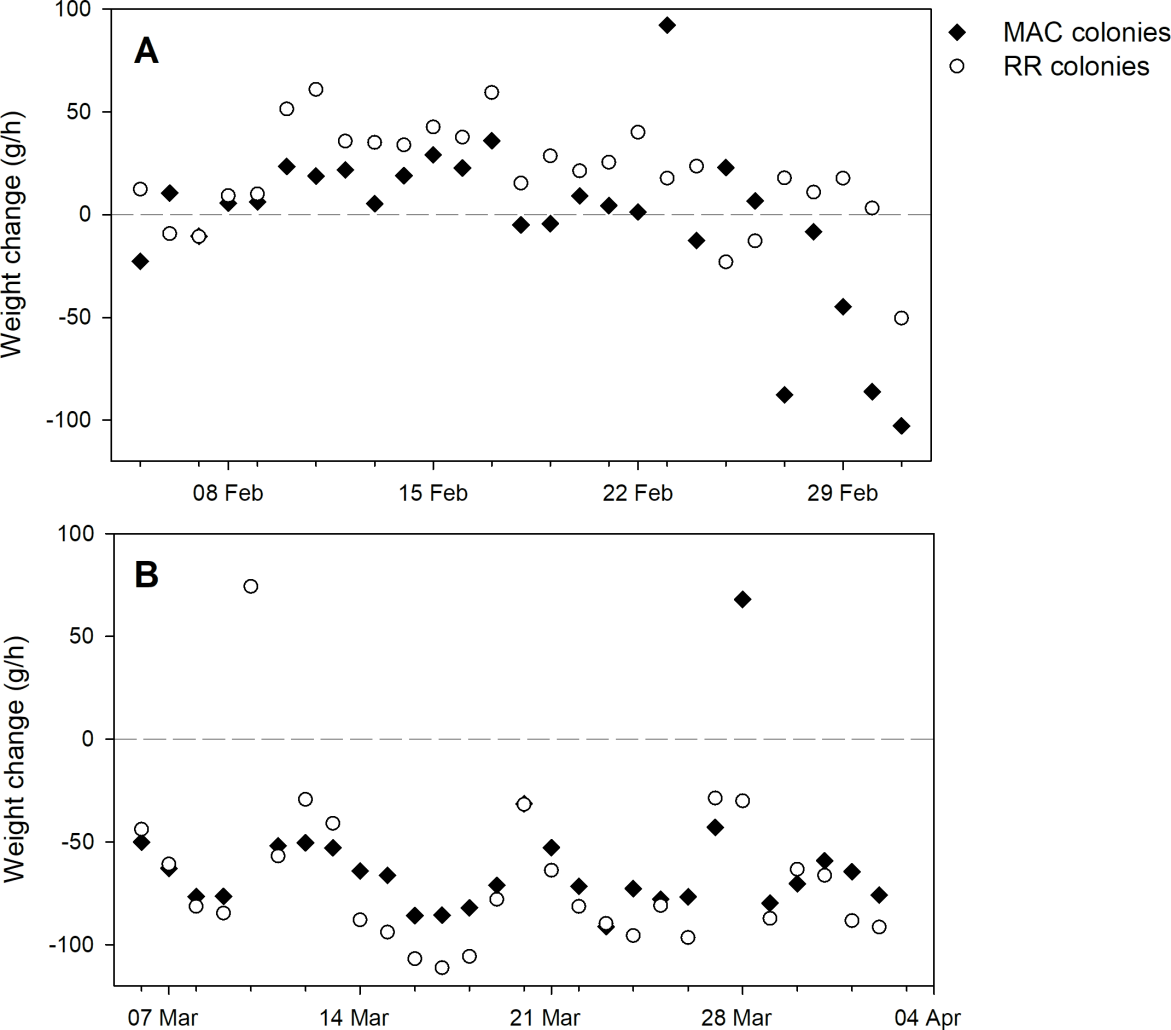
Average slope values of segments from piecewise regression curves fit to within-day weight change data, collected every 15 minutes, obtained from hives placed in proximity to forage (RR) and with little available forage (MAC) prior to exposure to blooming almonds followed by placement in unmanaged fields (the CAL 2015–16 dataset). A) slope of the regression segment just prior to the dusk break point; B) slope of the regression segment just after the dawn break point.

No differences were observed between treatment groups with respect to any parameter before almond pollination. However, during almond pollination the slope of the last segment before dusk was significantly higher among colonies that had access to forage compared to colonies that did not (Table 3; Fig 8). The slope of that segment would correspond to the change in hive mass due to returning foragers with nectar and pollen. No other parameters were significantly different between groups during pollination. After almond pollination, only the first segment after dawn, corresponding to weight loss due to forager departure, was significant.

**Table 3:** Results of a repeated measures MANOVA conducted on parameters of piecewise regressions fit to continuous hive weight data collected during and after almond pollination in California.

Dataset	Response variable	Factor	Num DF	Den DF	F Value	Pr > F
CAL 2015–16	4^th^ segment slope	treat	1	105.1	9.99	0.0021
(2 Feb.-3 Mar.)		day	26	278.6	2.70	<0.0001
		day*treat	26	278.6	1.40	0.0987
CAL 2015–16	2^nd^ segment slope	treat	1	168.5	7.06	0.0087
(6 Mar.-4 Apr.)		day	29	458.3	8.29	<0.0001
		day*treat	28	453.9	0.89	0.6341

## Discussion

Monitoring the weight honey bee hives continuously provides information, without colony disturbance, on how the hive is interacting with the environment. One of the challenges in the analysis of such data is modeling the data in a way that exploits the information in the dataset while reducing as much as possible the impact of spurious error. Running average weight data and detrended within-day data have been related to colony parameters such as total adult bee mass and foraging activity [5,6], and the methods they used to detrend and model the data (subtract raw hourly data from the 25 hour running average and fit sine curves to the result) provided robust estimates of bee flight activity. However, information was also lost due to the use of hourly averaging and of 3-day data subsets. In this study raw data were detrended by subtracting the value at midnight, rather than the running average, from each of the values of that day until the next midnight. A piecewise regression model was fit to single-day datasets in the original time scale using an R function [35] that is based on a bootstrapping method [36]. The user provided estimated break points, and the function used a random number generator to provide and test model parameters. The parameters were automatically adjusted until the fit reached a given threshold.

In order to reduce noise in the exploratory phase of model evaluation, daily detrended data were averaged overall 3–8 colonies in two independent datasets from different sites in the southwestern U.S. The resulting daily patterns were, for the most part, consistent among colonies within site. Because of the high precision and the random number algorithm in the segmented function to search for break points, parameters for a given fit were rarely identical, so 100 iterations of the function were conducted for each daily dataset and parameters for the best fit line, based on r^2^ value, were retained. Models with 3, 4 and 5 break points were evaluated and those with 4 break points offered a significantly higher r^2^ than those with 3 break points while often fitting more datasets than those with 5 break points. Models with 4 break points offered an alternative break point in the event the 1^st^ or 4^th^ break points were due to chance events unrelated to the study, such as a weather event. The function established break points where the change in the linear slope exceeded a predetermined threshold so providing an excessive number of break points resulted in a failure of model fit. Piecewise regression models in general fit the data well, with r^2^ values for regressions with 4 break points on average exceeding 0.97.

Having established that the piecewise regression method fit the data well, two experiments were conducted to test the interpretation of two parameters: the break point and following segment associated with initial forager departure in the morning (usually the first break point and second segment). The first experiment involved blocking the departure of foragers, without blocking gas or temperature exchange, just prior to dawn and keeping them blocked for fixed times. Models fit to the within-day weight data for each hive were used to determine the times of the initial forager departures. The results were consistent and the regression coefficient showed that for every hour the hive was blocked, the forager departure was delayed by about 40 minutes. The lack of a strict correspondence between planned and observed delay was likely due to factors such as the inherent variability in forager departure (particularly outside of a nectar flow) and to hive weight change resulting from moisture loss due to decreasing ambient r.h. after dawn, rather than bee movement. The study did confirm that the dawn break point resulted largely from an increased loss of hive mass due to forager departure. The second experiment involved monitoring bee traffic for one minute in the morning, during the period of time associated with the initial forager departure. Net bee loss (i.e. more departures than arrivals) during that minute was significantly related to the slope of the piecewise regression model associated with that time of the morning. The value of that slope was a function of the numbers and masses of arriving (laden) and departing (unladen) bees, and was used to estimate an average payload of arriving bees of about 0.02 g per bee. The better fit of piecewise regression lines with 5 break points to data from Australia compared to 4 break points, which fit Arizona data better, suggested that the optimal number of break points for such analyses may depend on the environment.

The piecewise regression approach was then used to analyze data from an experiment on bee colony nutrition conducted in Arizona and California. Within-day data from hives from two groups, one that had been exposed to abundant forage and one that had been exposed to little forage, were compared with respect to nine regression parameters (4 break points and 5 segment slopes). Neither hive evaluation data nor sine-based models of within-day weight data revealed group differences during any of the time periods (i.e. during treatment, during almond pollination, and post pollination), and analyses of bee gut microbiota did not reveal significant differences [34]. However, the regression approach did detect a higher rate of weight gain during forager return (usually the 4^th^ segment) during almond pollination, indicating that the well-nourished colonies had greater foraging success even though no significant differences were observed in the mass of foragers. Similarly, during the post almond period, only the forager departure slope was significantly different between groups, indicating that the well-nourished hives likely either produced more foraging bees, or caused increased foraging effort due to factors such as increased demand, even though forage opportunities at that time and location were poor for all hives (and there was no difference between groups in foraging success, as shown by the lack of a difference in the slopes of segments associated with forager return). In the analysis presented here, no significant differences were detected with respect to break point timing, suggesting that the nutritional state did not affect timing of colony activity.

Within-day weight changes over one to 15 minutes from hives at different locations and years showed consistent patterns that had biological interpretations, that were sensitive to environmental factors such as the presence of a nectar flow, and that could be exploited using piecewise regression. Analyses of within-day data from a field experiment involving colony nutrition detected colony-level treatment differences where other methods, such as visual inspections, did not. In addition, the data were collected without colony disturbance, which can be an important, particularly during cold weather. These results demonstrate the potential of continuous monitoring of honey bee hives as a means of providing easily-interpreted colony-level response variables for longitudinal field experiments.

## Conclusions

- The slopes and break points of segmented lines fit to detrended continuous hive weight data using piecewise regression provided information on colony behavior;
- Piecewise regression lines usually had break points after sunrise, indicating the start of daily colony activity, and before sunset, indicating the end of daily colony activity;
- The interpretation of the break point and following segment associated with the initiation of daily hive activity were confirmed by: 1) manipulating forager departure times by closing the entrance for variable periods of time; and 2) monitoring bee traffic;
- The piecewise regression analysis of detrended within-day weight data were applied to data from fed and starved bee colonies during and after almond pollination in California, and the regression indicated that the fed colonies had greater foraging success during almonds, although the initial rates of mass loss due to forager departures were not different; the opposite was true during a pollen dearth after almond pollination.

## Supporting information

S1 Table Entrance closing schedule to manipulate the start of flight activity. (PDF)

S1 Fig Sample daily data files from an experiment involving manipulation of time of initial forager departure by blocking the entrance. Delays were calculated with respect to 5:30AM (about dawn). Black line: average within-day weight change of Block 1 hives (gray shaded area shows s.e.); blue line: average within-day weight change of Block 2 hives (blue shaded area shows s.e.).
(PDF)

S2 Fig Comparison of planned delay in initial forager departure with observed delay. Data show 1^st^ break point of Block 1 regression minus 1st break point of Block 2 regression. Regression equation: y = 0.649x-0.225.
(PDF)

S3 Fig Regression of segment slope, from a piecewise regression fit to within-day hive weight change, on net bee movement (arrivals–departures) over one minute for hives in Sydney, Australia. Solid line is regression. Regression equation: y = 0.032x-1.1866.
(PDF)

S1 File **Figs A-D**. Piecewise regression lines (solid lines) fit to within-day weight changes collected every 5 minutes for 16 hives on 29 March, and 20, 21 and 24 April, 2017, in Sydney, Australia.
(PDF)

S2 File Experimental data. (XLSX)
