# Supplementary material for: Using within-day hive weight changes to measure environmental effects on honey bee colonies
Source: PLoS One. 2018 May 23;13(5):e0197589. doi: 10.1371/journal.pone.0197589 (PMC5965838; doi:10.1371/journal.pone.0197589)
Supplement: S1 Table — (PDF) [file pone.0197589.s001.pdf]

S1 Table. Entrance closing schedule to manipulate the start of flight activity. Block 1 consisted of 3 hives and Block 2 consisted of 4 hives, and "delay" pertains to the length of time the gates were closed starting at 5:30AM.

		Block 1		Block 2	
Date	Day	Delay	Time Open	Delay	Time Open
8/12/2015	2	0:00	Open	2.5 hr	8:00
8/13/2015	3	1 hr	6:30	0:00	Open
8/14/2015	4	0:00	Open	2 hr	7:30
8/17/2015	7	1.5hr	7:00	0:00	Open
8/18/2015	8	0:00	Open	1.5 hr	7:00
8/19/2015	9	2 hr	7:30	0:00	Open
8/20/2015	10	0:00	Open	3 hr	8:30
8/21/2015	11	2 hr	7:30	0:00	Open
8/24/2015	14	0:00	Open	3 hr	8:30
8/25/2015	15	1.5 hr	7:00	0:00	Open
8/26/2015	16	0:00	Open	2 hr	7:30
8/27/2015	17	2 hr	7:30	0:00	Open
8/28/2015	18	0:00	Open	2 hr	7:30
