# Supplementary material for: Using within-day hive weight changes to measure environmental effects on honey bee colonies
Source: PLoS One. 2018 May 23;13(5):e0197589. doi: 10.1371/journal.pone.0197589 (PMC5965838; doi:10.1371/journal.pone.0197589)

S1 Figure. Sample daily data files from an experiment involving manipulation of time of initial forager departure by blocking the entrance. Delays were calculated with respect to 5:30AM (about dawn). Black line: average within-day weight change of Block 1 hives (gray shaded area shows s.e.); blue line: average within-day weight change of Block 2 hives (blue shaded area shows s.e.).

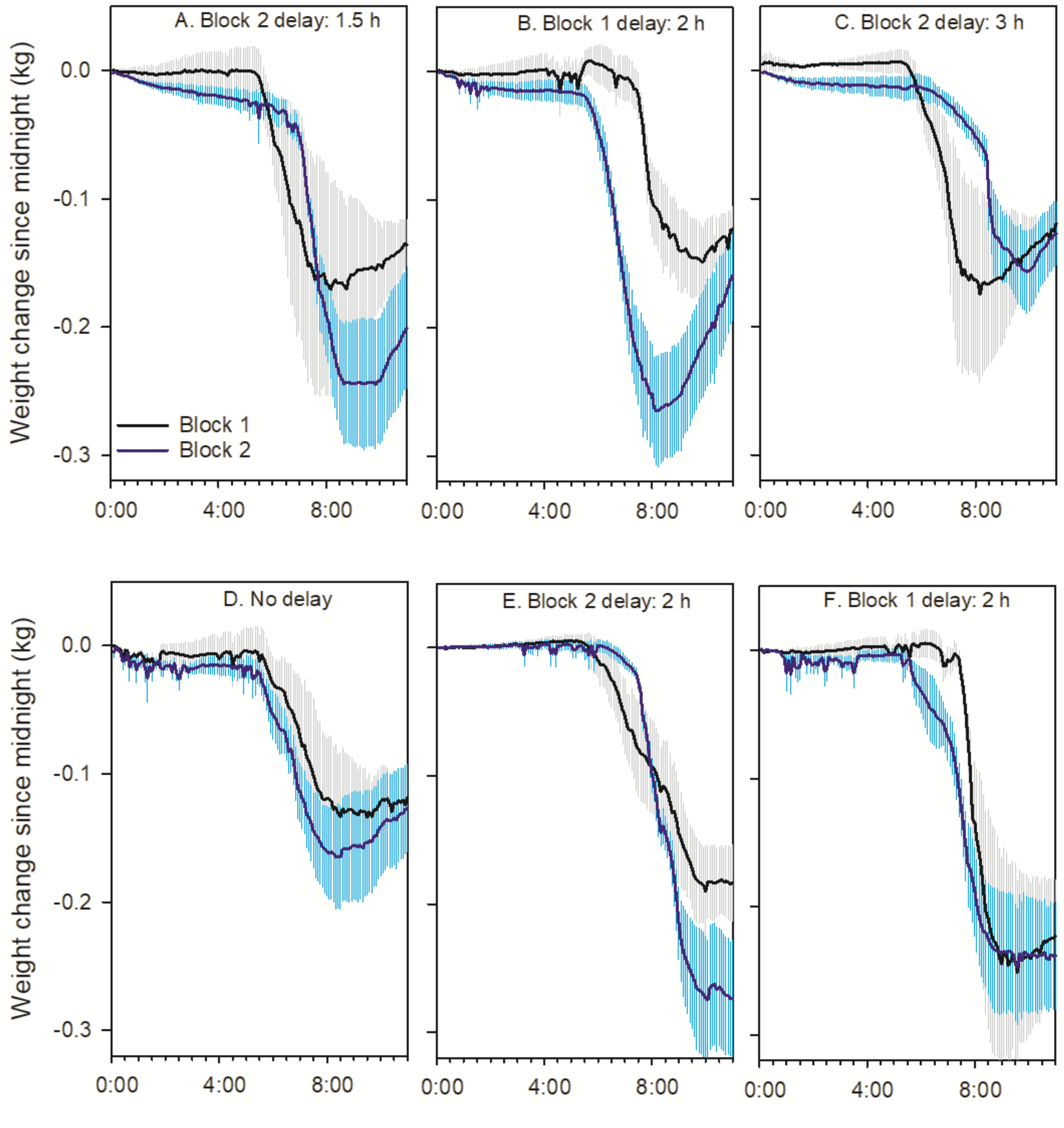

Supplement: S1 Fig — Delays were calculated with respect to 5:30AM (about dawn). Black line: average within-day weight change of Block 1 hives (gray shaded area shows s.e.); blue line: average within-day weight change of Block 2 hives (blue shaded area shows s.e.). (PDF) [file pone.0197589.s002.pdf]
