# Supplementary material for: Using within-day hive weight changes to measure environmental effects on honey bee colonies
Source: PLoS One. 2018 May 23;13(5):e0197589. doi: 10.1371/journal.pone.0197589 (PMC5965838; doi:10.1371/journal.pone.0197589)

**S2 Figure.** Comparison of planned delay in initial forager departure with observed delay. Data show 1st break point of Block 1 regression minus 1st break point of Block 2 regression. Regression equation: y=0.649x-0.225.

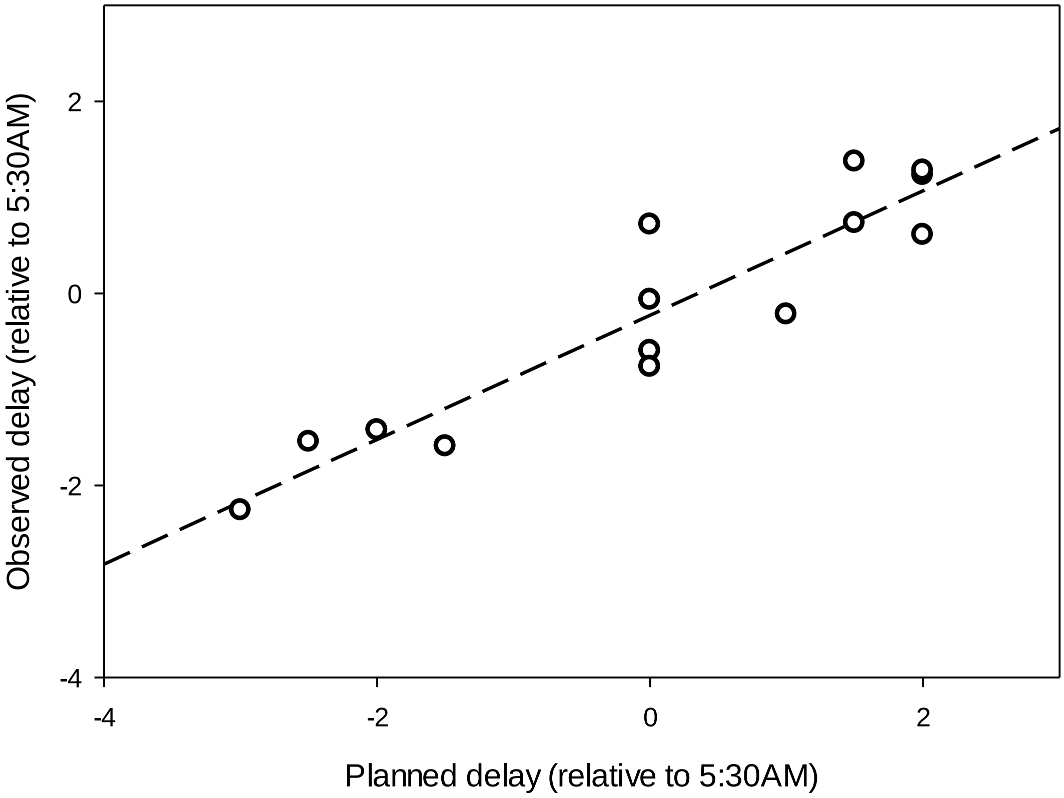

Supplement: S2 Fig — Data show 1st break point of Block 1 regression minus 1st break point of Block 2 regression. Regression equation: y = 0.649x-0.225. (PDF) [file pone.0197589.s003.pdf]
