# Supplementary material for: Using within-day hive weight changes to measure environmental effects on honey bee colonies
Source: PLoS One. 2018 May 23;13(5):e0197589. doi: 10.1371/journal.pone.0197589 (PMC5965838; doi:10.1371/journal.pone.0197589)

**S3 Fig.** Regression of segment slope, from a piecewise regression fit to within-day hive weight change (g), on net bee movement (arrivals – departures) over one minute for hives in Sydney, Australia. Solid line is regression. Regression equation: y =0.032x-1.1866.

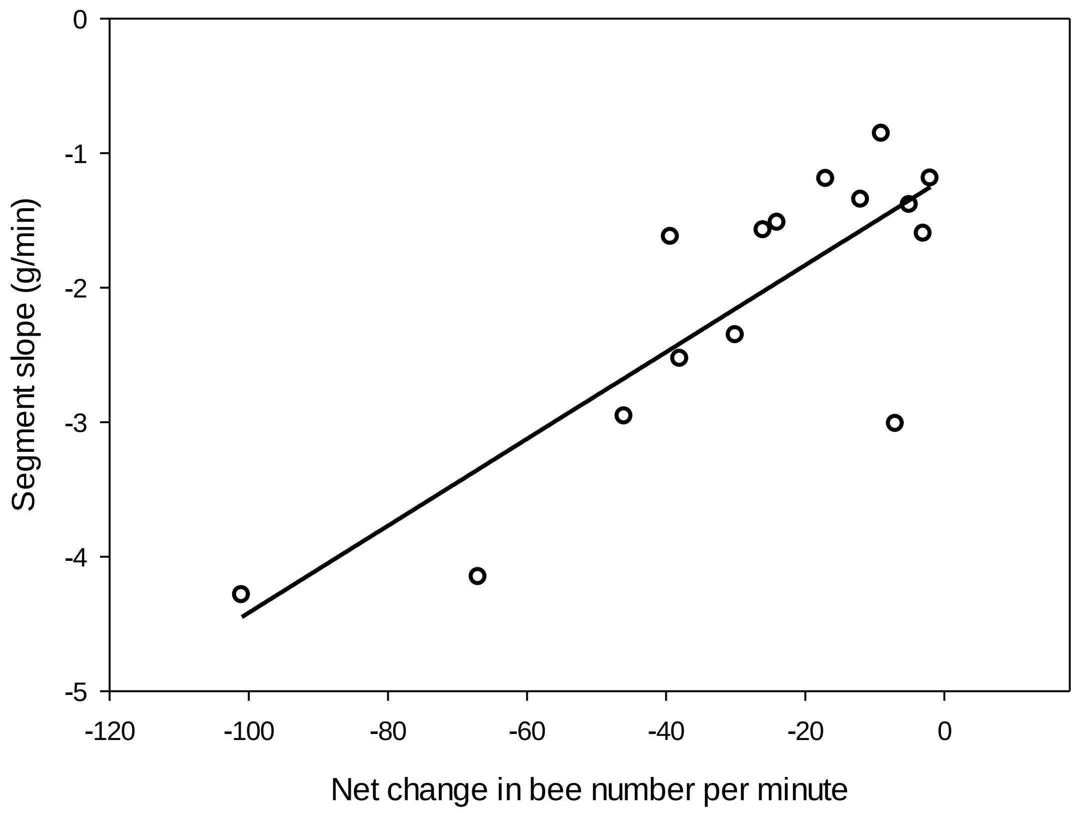

Supplement: S3 Fig — Solid line is regression. Regression equation: y = 0.032x-1.1866. (PDF) [file pone.0197589.s004.pdf]
