# Supplementary material for: Using within-day hive weight changes to measure environmental effects on honey bee colonies
Source: PLoS One. 2018 May 23;13(5):e0197589. doi: 10.1371/journal.pone.0197589 (PMC5965838; doi:10.1371/journal.pone.0197589)

Figure A. Piecewise regression lines (solid lines) fit to within-day weight changes collected every 5 minutes for 4 hives on 29 March 2017 in Sydney, Australia.

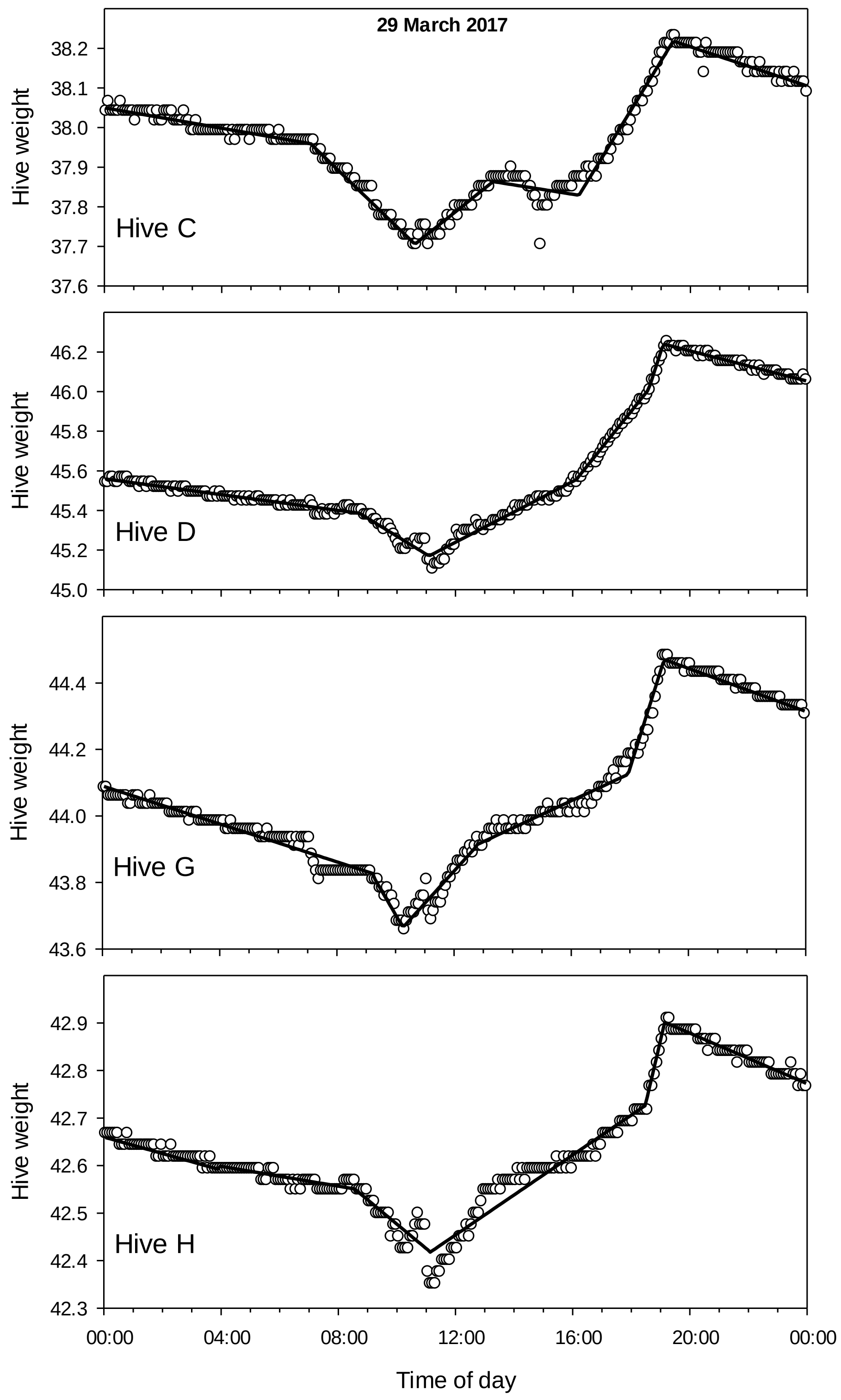

Figure B. Piecewise regression lines (solid lines) fit to within-day weight changes collected every 5 minutes for 4 hives on 20 April 2017 in Sydney, Australia.

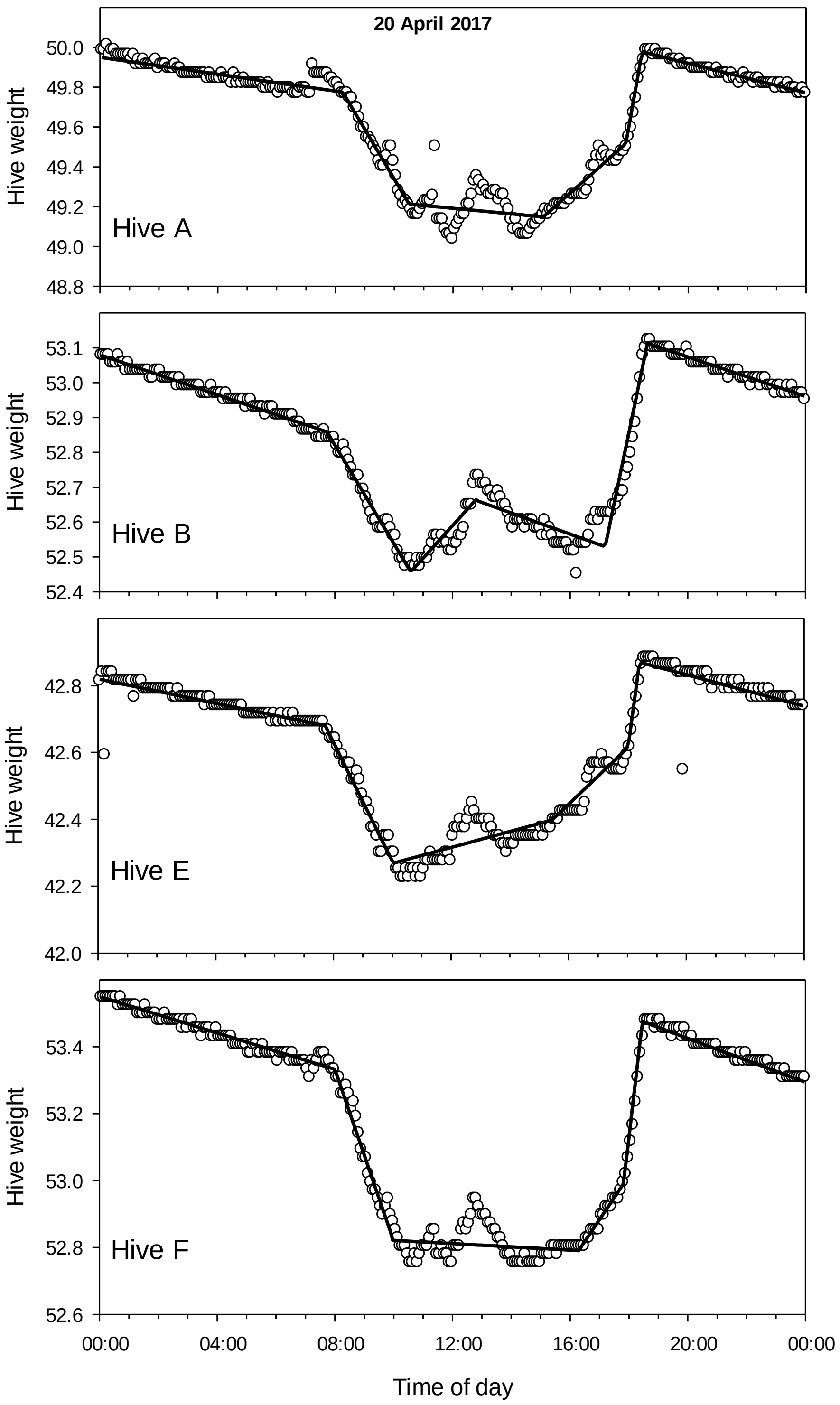

Figure C. Piecewise regression lines (solid lines) fit to within-day weight changes collected every 5 minutes for 4 hives on 21 April 2017 in Sydney, Australia.

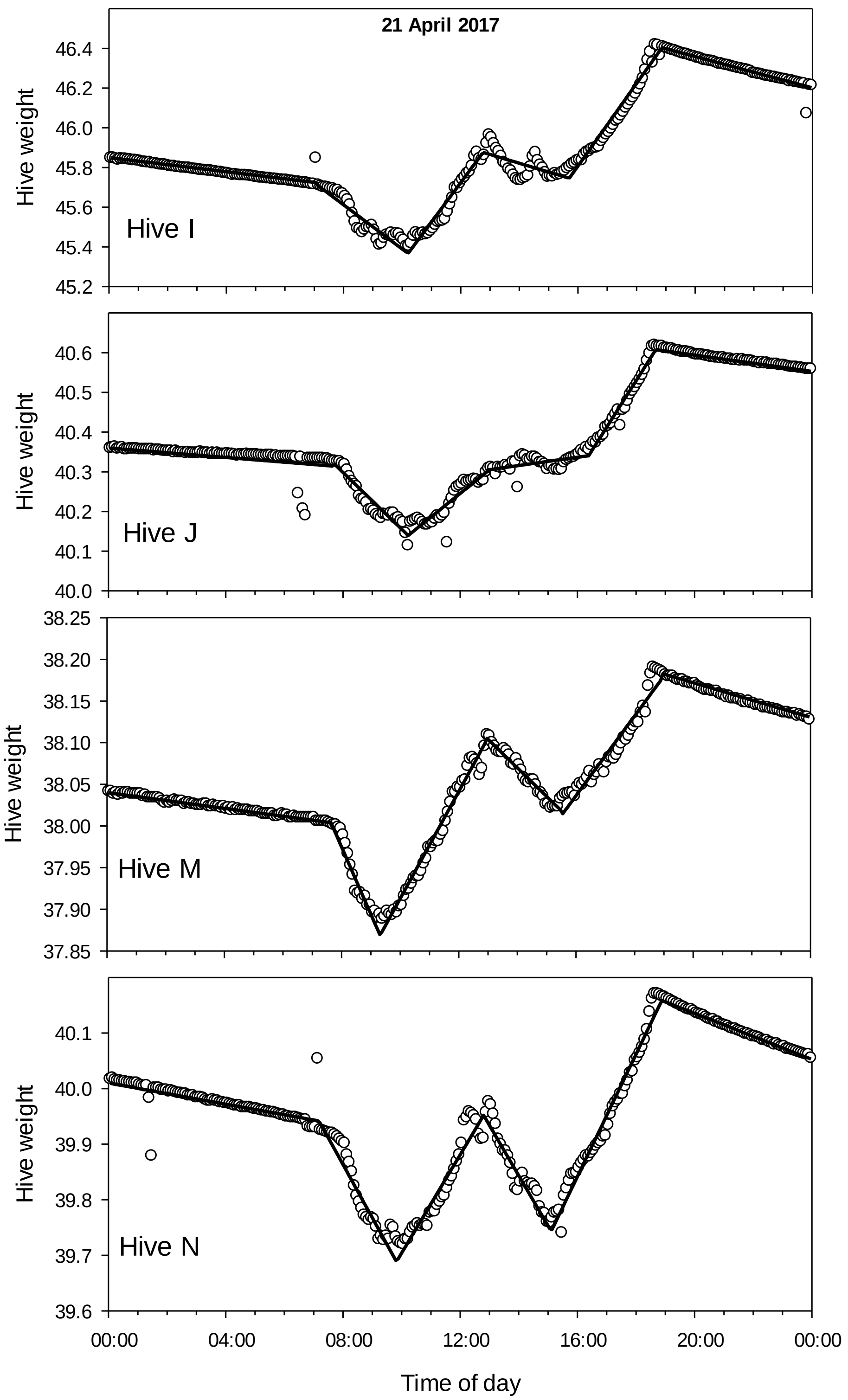

Figure D. Piecewise regression lines (solid lines) fit to within-day weight changes collected every 5 minutes for 4 hives on 24 April 2017 in Sydney, Australia.

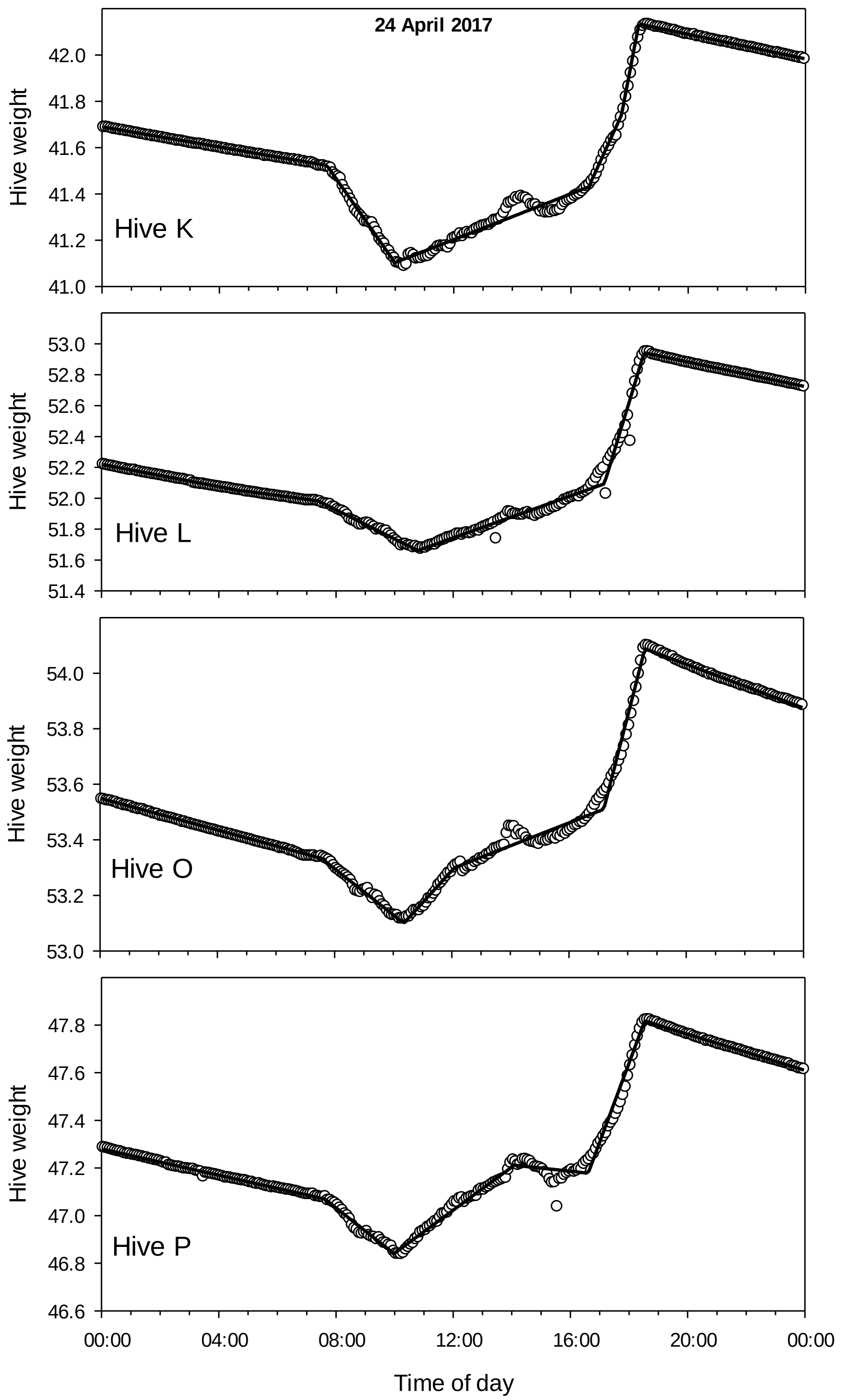

Supplement: S1 File — Figs A-D. Piecewise regression lines (solid lines) fit to within-day weight changes collected every 5 minutes for 16 hives on 29 March, and 20, 21 and 24 April, 2017, in Sydney, Australia. (PDF) [file pone.0197589.s005.pdf]
